# Maternal Uterine Artery Doppler and Serum Marker in the First Trimester as Predictive Markers for Small for Gestational Age Neonates and Preeclampsia: A Pilot Study

**DOI:** 10.3390/diagnostics15020233

**Published:** 2025-01-20

**Authors:** Je Yeon Lee, Kyung A Lee, So Yun Park, Soo Jung Kim, So-Yeon Shim, Young Ju Kim, Mi Hye Park

**Affiliations:** 1 Department of Obstetrics and Gynecology, Ewha Medical Center, Ewha Medical Institute, Ewha Womans University College of Medicine, Seoul 07804, Republic of Korea; marienssono@gmail.com (J.Y.L.); leekyunga@gmail.com (K.A.L.); obgypsy@ewha.ac.kr (S.Y.P.); bossksj25@gmail.com (S.J.K.); kkyj@ewha.ac.kr (Y.J.K.); 2 Department of Pediatrics, Ewha Medical Center, Ewha Medical Institute, Ewha Womans University College of Medicine, Seoul 07804, Republic of Korea; simso@ewha.ac.kr

**Keywords:** small for gestational age, preeclampsia, uterine artery doppler, first trimester, uterine artery notch, beta-hCG, PAPP-A

## Abstract

**Background/Objectives**: Although preeclampsia (PE) and small for gestational age (SGA) are known to come from impaired placentation during the first trimester, prior studies have focused mostly on Doppler findings in the second trimester. **Methods**: In this retrospective pilot study, we enrolled 628 singleton pregnant women who underwent ultrasound in both the first and second trimesters and blood test. For SGA correlation, we further excluded 12 subjects with PE because PE may be the cause of SGA. We first presented the reference range of parameters of uterine artery Doppler in the first trimester and then grouped the subjects according to the presence of SGA (presence = 104, absence = 512) or PE (presence = 12, absence = 616) and investigated the association of uterine artery Doppler findings and serum markers in the first trimester with the occurrence of SGA or PE. **Results**: The uterine artery pulsatility index and the resistance index and the proportion of uterine artery notch decreased progressively in the first trimester. A lower serum beta-hCG level in the first trimester predicted the occurrence of SGA (adjusted odds ratio [AOR] = 0.53, *p* = 0.019), while the presence of the uterine artery notch in the first trimester predicted the development of PE (notch at least on one side: AOR = 8.65, *p* = 0.045 and notch on both sides: AOR = 8.91, *p* = 0.047). Regardless of whether a notch was present in the second trimester, a uterine artery notch in the first trimester was associated with an excellent negative predictive value (99.6%) for PE. **Conclusions**: This study suggests the clinical importance of assessing serum beta-hCG and the uterine artery notch in the first trimester to predict SGA and PE.

## 1. Introduction

Ultrasound was introduced to the medical field at least 50 years ago and has remained a useful tool for evaluating fetal and maternal conditions antenatally and during emergency prenatal care [1]. Unlike other diagnostic tools, such as magnetic resonance imaging and computed tomography, the sonographic approach is easy and fast. Recently, Doppler sonography for ultrasound imaging for blood flow has been increasingly utilized for the evaluation of preeclampsia (PE) and other adverse perinatal outcomes, such as small for gestational age (SGA) [2,3,4].

PE affects 3% of all pregnancies and is associated with high maternal prenatal mortality and morbidity [5]. For this reason, many researchers have focused on predicting and evaluating PE [5,6]. PE is known to result from impaired placentation during the first trimester. Likewise, SGA is also associated with defective placentation [7] and can lead to perinatal mortality and morbidity and poorer short- and long-term health outcomes [8,9]. When the trophoblast invades the spiral arteries between 8 and 12 weeks in utero, placental remodeling begins, and defects during this time can lead to SGA [10]. Although Doppler ultrasound can provide information on the perfusion of uteroplacental circulation in the first trimester, prior research has focused mostly on Doppler findings in the second trimester [11].

Previous studies that have investigated the clinical role of the Doppler in the first trimester in predicting SGA or PE have produced heterogenous results [12,13]. A high mean pulsatility (PI) index of uterine artery was reported to have high negative predictive value and specificity for predicting SGA or PE [14], while another study showed that a high resistance index (RI) had high negative predictive value and specificity only for SGA [15]. Moreover, the predictive value of bilateral uterine artery notches on pregnancy-related outcomes showed conflicting results [15,16]. Inconsistent results of Doppler finding in the trimester for predicting SGA or PE have prevented clinicians from using it as a routine screening test during gestation.

Several studies elucidated the association between serum biomarkers, such as pregnancy-associated plasma protein A (PAPP-A), a disintegrin and metalloprotease 12, placental growth factor (PlGF), soluble fms-like tyrosine kinase 1, placental protein 13, free beta-human chorionic gonadotrophin (beta-hCG), with PE and SGA in the first trimester [6,17,18,19,20,21]. Furthermore, the accuracy in detecting PE and SGA was increased by combining uterine artery Doppler findings with serum biomarkers [22,23,24,25]. A high detection rate (93.1%) of early PE was reported with the combination of maternal history, uterine artery pulsatility index (PI), mean arterial pressure, PAPP-A, and PlGF [26,27]. Despite the high accuracy, some serum markers except for beta-hCG and PAPP-A which are routinely screened for Down syndrome are expensive and cannot be routinely analyzed in some countries.

In this pilot study, we hypothesized that the Doppler findings of the uterine artery in the first trimester would vary with the gestational age during placentation and that routine serum markers and Doppler findings would have unique predictive value for SGA and PE. Therefore, we first presented the reference range for the parameters of uterine artery Doppler in the first trimester and then, investigated the usefulness of serum levels of beta-hCG and PAPP-A and uterine artery Doppler findings in predicting SGA and PE.

## 2. Materials and Methods

### 2.1. Study Subjects

**Figure 1:**
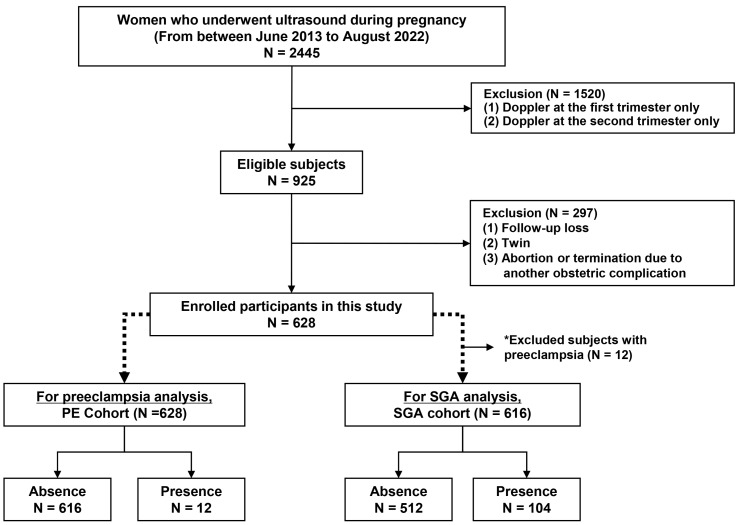
Flowchart of the study participants and enrollment. * A total of 12 subjects with preeclampsia among the 628 subjects were excluded for SGA analysis because PE itself is the major cause of SGA. Abbreviations: SGA, small for gestational age.

We retrospectively reviewed the Ewha Mother and Baby Center database of pregnant women who were admitted to the Ewha Womans University Medical Center, Seoul, Korea, between June 2013 and August 2022. A total of 2455 pregnant women underwent routine ultrasounds including uterine artery Doppler recording at least once during pregnancy (Figure 1). The exclusion criteria were as follows. (1) Subjects who underwent ultrasound only once in the first or second trimester. (2) Subjects with twin pregnancies. (3) Abortion or termination due to another obstetric complication. (4) Loss of follow-up or missing Doppler findings at either the first or second trimester. After excluding 1827 women with data, 628 were included in the final analysis. For SGA correlation, we further excluded 12 subjects with PE among the 628 subjects to see the independent effect of SGA on Doppler findings without the effect of PE because PE is the major cause of SGA. Therefore, the total number of enrolled subjects to group SGA was 616 and those to group PE was 628. Gestational age was calculated from the last menstruation and confirmed by a crown–rump length measurement. Premature delivery was defined when gestational age at birth was before 37 completed weeks.

This study was approved by the Ewha Womans University Medical Center institutional review board. The need for informed consent was waived because of the retrospective nature of this study.

### 2.2. Maternal Serum Markers and Sonographic Procedures

Maternal non-fasting blood samples were collected using a serum separation tube (4 mL each) for measuring beta-hCG and PAPP-A levels as a routine screening for Down syndrome between 10 and 13 weeks of gestational age. The serum samples were immediately centrifuged and stored at 2 °C to 8 °C. On the following day, samples were delivered to the Seegene Medical Foundation (Seoul, Republic of Korea) and analyzed by fluoroimmunoassay using an automated AutoDelfia system (Perkin Elmer Brazil, Wallac, Turku, Finland). Both serum markers were expressed as multiples of the median (MoM), adjusted for the gestational age.

**Figure 2:**
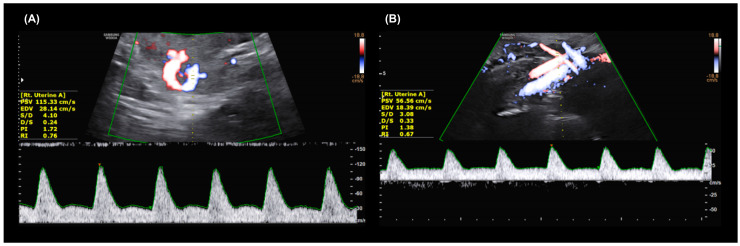
Representative transabdominal Doppler images of the uterine artery (**A**) in the first trimester (10–13 weeks) with an early diastolic notch and (**B**) in the second trimester (20–26 weeks) with normal uterine artery waveforms.

Bilateral uterine artery Doppler was conducted twice during routine ultrasound examinations, in the first (10–13 weeks) and second (20–26 weeks) trimesters. Ultrasonographic examination was performed by one expert obstetrician using a VolusonTM Expert 8 BT18 (GE Healthcare, Waukesha, WI, USA) and Expert 10 BT19 (GE Healthcare, Waukesha, WI, USA) ultrasound machine with an electronic matrix 4D Convex abdominal probe of 1.0–6.0 MHz. In the first trimester, the transabdominal transducer was tilted laterally from the mid-sagittal view of the cervix until both uterine arteries were identified by color Doppler at the point just before the uterine artery branches into the arcuate artery (Figure 2A). In the second trimester, the transabdominal probe was placed in the para-uterine area at the inguinal ligament and toward the point where the uterine artery crosses over with the external iliac artery by color Doppler (Figure 2B) [28]. The angle between the transducer beam and the targeted vessel was less than 60°. The PI and the resistance index (RI) were measured at each right and left side using more than three adequate consecutive Doppler waveforms and the average (mean) was recorded. Early diastolic notches on each side were also examined and recorded as absent or present (right, left, or both sides). We defined the existence of a persistent notch when a notch is continuously found on the same side from the first and second trimester Doppler sonography. We classified the subject into four groups according to the presence of the notch in the first and second trimesters. UAN1st_−_2nd_−_ group = no uterine artery notch in both the first and second trimesters (*n* = 267). UAN1st_+_2nd_−_ group = present uterine artery notch in the first trimester but not in the second trimester (*n* = 330). UAN1st_−_2nd_+_ group = no notch in the first trimester but a notch in the second trimester (*n* = 1). UAN1st_+_2nd_+_ group = the uterine artery notch found in both the first and second trimesters, indicating the persistent notch (*n* =30).

### 2.3. Clinical Definition for the Diagnosis

SGA was defined as birth weight for gestational age < 10th percentile according to the cohort of live births chart [29]. Percentile was measured based on the gender-specific reference population charts from the World Health Organization (WHO) [30,31]. Importantly, subjects with PE were excluded from SGA analyses because PE itself can be a direct cause of SGA.

PE was defined as follows [32]: systolic blood pressure ≥ 140 mmHg or diastolic blood pressure ≥ 90 mmHg for the first time after 20 weeks, with proteinuria defined as ≥300 mg in 24 h urine, urine protein to creatinine ratio ≥ 0.3, or 1+ dipstick protein on the dipstick in a random urine sample. In the absence of proteinuria, PE was defined as newly onset hypertension with the new onset of any of the followings: thrombocytopenia as platelets < 100,000/μL; insufficient renal function as creatinine > 1.1 mg/dL or more than twice the baseline; liver involvement as serum transaminase twice the normal level; neurological symptoms such as headache, visual disturbances, and convulsions; and pulmonary edema. All subjects with PE in this study had proteinuria which met the above clinical criteria. A total of 1 case had oligohydramnios, 1 case had placenta abruptio and 6 cases gave birth prematurely. NO HELLP syndrome was found in all cases. Depending on the time of onset, 7 cases were developed PE late (≥34 wks), while 5 cases were developed PE early (<34 wks). Three cases had severe features according to the operational definition [33].

### 2.4. Statistical Analysis

Baseline demographic characteristics were analyzed and compared. For categorical variables, the chi-square tests or Fisher’s exact tests were used. For continuous variables, the distribution was first examined for normality using the Kolmogorov–Smirnov test. When data did not deviate from normal distribution, the independent *t*-tests were applied for the group comparison. When data were not normally distributed, Mann–Whitney *U* tests were applied for the group comparison. The association of the uterine artery notch between the first and second trimesters and the proportions of the uterine artery notch in the first trimester according to the presence of SGA, PE, or uterine artery notch were compared using the chi-square tests or Fisher’s exact tests. Sensitivity was measured as the fraction of those with the uterine artery notch in the first trimester among those with the uterine artery notch in the second trimester, SGA, or PE. Specificity was the fraction of those without the uterine artery notch in the first trimester among those without the uterine artery notch in the second trimester, SGA, or PE. Positive predictive value was the fraction of those with the uterine artery notch in the second trimester, SGA, or PE who had uterine artery notch in the first trimester. Negative predictive value was the fraction of those without the uterine artery notch in the second trimester, SGA, or PE who had no uterine artery notch in the first trimester. Logistic regression analysis was used to investigate the Doppler or serum parameters associated with the development of SGA or PE, after controlling for maternal age, gestational age, hypertension, autoimmune disease, gestational diabetes mellitus, nulliparity, and body mass index. Data were analyzed using SPSS software, ver. 25 (IBM Corporation, Armonk, NY, USA). P-values less than 0.05 were considered significant.

## 3. Results

### 3.1. Maternal Demographic Characteristics According to the Presence of SGA and PE

The demographic characteristics and pregnancy outcomes of the groups are shown in Table 1. There were no differences in age, body mass index, chronic hypertension, diabetes mellitus, autoimmune disease, gestational diabetes mellitus, premature delivery, a poor neonatal outcome assessed using 1 min and 5 min Apgar scores between the SGA and non-SGA groups and between the PE and non-PE groups. The PE group had a higher proportion of gestational hypertension than the non-PE group. The PE group delivered earlier than the non-PE group, while there was no difference between SGA and non-SGA groups. The proportion of SGA was significantly higher in nulliparous mothers than in parous mothers. The SGA group had significantly lower levels of beta-hCG than the non-SGA group and no difference in the PAPP-A level between the two groups. There were also no differences in serum beta-hCG and PAPP-A levels regardless of the presence of PE.

### 3.2. Serum and Doppler Parameters in the First Trimester

**Figure 3:**
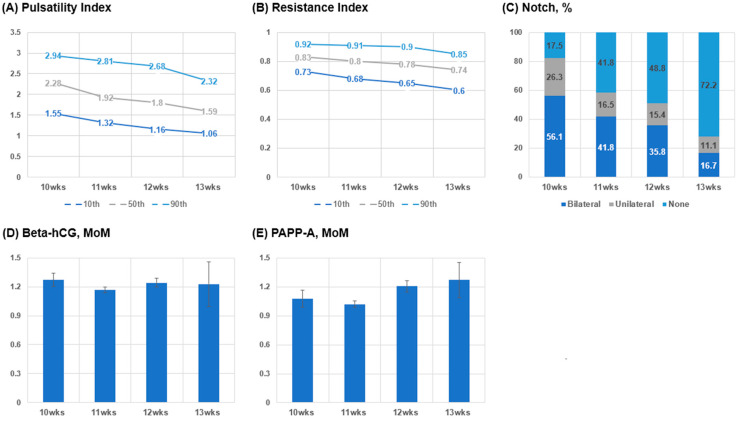
The Doppler findings and serum biomarkers in the first trimester according to gestational age. Abbreviation: MoM, multiples of the median. (**A**) Pulsatility Index; (**B**) Resistance Index; (**C**) Notch, %; (**D**) Beta-Hcg, MoM; (**E**) PAPP-A. MoM.

We explored the 10th, 50th, and 90th percentiles of uterine artery PI and RI, and the proportion of uterine artery notch according to the gestational age at which the Doppler was performed in all subjects (Figure 3 and Table S1). The 10th, 50th, and 90th percentiles of the uterine artery PI and RI values were inversely related to an increasing gestational age from 10 to 13 weeks (Figure 3A,B). The proportion of uterine artery notch was decreased from 10 to 13 weeks (Figure 3C). At 10 weeks, 82.5% of subjects presented with a uterine artery notch (unilateral notch, 26.3% and bilateral notch, 56.1%) while 27.8% of subjects presented with a notch at 13 weeks (unilateral notch, 11.1% and bilateral notch, 16.7%). There was no difference in the proportion of unilateral and bilateral notches from 10 to 13 weeks. Regarding the serum markers, serum levels of beta-hCG and PAPP-A were not significantly different across the gestational ages examined (Figure 3D,E).

### 3.3. Association of the Uterine Artery Notch Between the First and Second Trimesters

**Figure 4:**
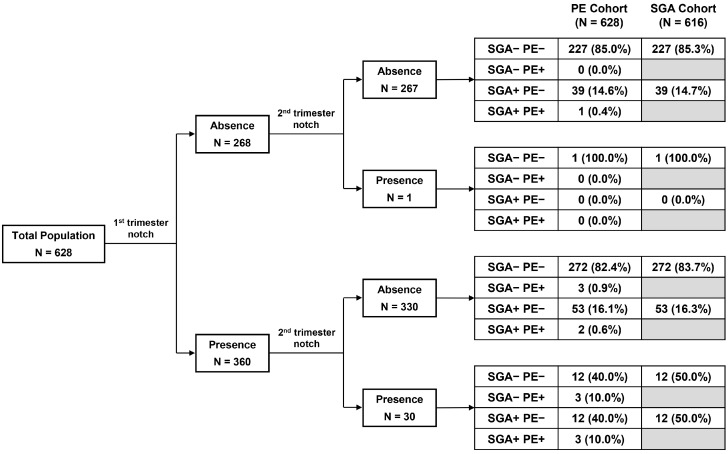
Flowchart of the association between the uterine artery Doppler findings in the first and second trimesters and the presence of SGA and PE. Abbreviations: PE, preeclampsia; SGA, small for gestational age.

The relationship of the uterine artery notch between the first and second trimesters is shown in Table 2 and Figure 4. Compared to those without the uterine artery notch in the first trimester, those with the uterine artery notch at least in one side in the first trimester had a higher proportion of the uterine artery notch in the second trimester and the persistent notch. Relative to the presence or absence of the uterine artery notch in the second trimester, the diagnostic performance of the uterine artery notch in the first trimester is as follows: sensitivity, 96.8%; specificity, 44.7%; positive predictive value 8.3%; and negative predictive value 99.6% (Table 2). That is, of the 268 subjects without a uterine artery notch in the first trimester, only one subject had a notch in the second trimester, meaning an excellent negative predictive value. Moreover, the one subject with a notch only in the second trimester did not experience SGA or PE. Of those 30 subjects who had uterine artery notches in both trimesters, 12 (40.0%) subjects had neither SGA nor PE, 12 (40.0%) had SGA only, 3 (10.0%) had PE only, and 3 (10.0%) had both SGA and PE. For the whole 628 subjects, the prevalence of a persistent notch was 4.8%.

We also subdivided the presence of the uterine artery notch into unilateral and bilateral (Table S2). Of the 106 subjects having a unilateral uterine artery notch in the first trimester, 4 subjects (3.8%) had a unilateral notch in both trimesters, and 2 subjects (1.9%) had bilateral uterine artery notches in the second trimester. Of the 254 subjects with bilateral uterine notches in the first trimester, 18 subjects (7.1%) had a unilateral uterine artery notch in the second trimester and 6 subjects (2.4%) had bilateral uterine artery notches in both trimesters. However, there was no difference in the proportion of unilateral and bilateral notches between the first and second trimesters. The prevalence of a persistent notch on bilateral sides was 1.0% among the whole 628 subjects.

### 3.4. Uterine Artery Notch According to the Presence of SGA and PE

The proportion of subjects with a uterine artery notch was not different between the SGA and non-SGA groups, while the notch was significantly more common in the PE group than the non-PE group in the first trimester (Table 3). In the second trimester, the proportion of subjects with a notch was significantly higher in the SGA or PE groups than in the non-SGA or non-PE groups. Both the SGA and PE groups had significantly higher proportion of subjects in group 4 than those in groups 1 and 2. The diagnostic performance of the uterine artery notch in the first trimester for the prediction of SGA and PE was as follows: sensitivity, 62.5% and 91.7%; specificity, 44.5% and 43.3%; positive predictive value, 18.6% and 3.1%; and negative predictive value 85.4% and 99.6%, respectively (Table 4).

### 3.5. Doppler and Serum Parameters Predicting SGA or PE

In the logistic regression analysis, no Doppler parameters in the first trimester predicted the presence of SGA, while lower levels of beta-hCG significantly predicted SGA (adjusted odds ratio [AOR] = 0.53, 95% CI 0.27–0.90; Table 5). On the contrary, a uterine artery notch at least on one side or on both sides significantly predicted PE (notch at least on one side: AOR = 8.65, 95% CI 1.06–77.77 and notch on both sides: AOR = 8.91, 95% CI 1.05–82.28). The other parameters did not predict PE. In the second trimester, the PI, the RI, a notch at least on one side, and a persistent notch predicted the presence of SGA, while all the uterine Doppler parameters predicted PE.

## 4. Discussion

In this study, we presented the uterine artery Doppler parameters according to the gestational age in the first trimester and investigated whether maternal serum markers and Doppler findings in the first trimester were related to SGA or PE. Our major findings are as follows. (1) The uterine artery PI and RI, and the proportion of subjects with a uterine artery notch decreased progressively in the first trimester, while the serum markers were not different across the gestational ages. (2) The serum beta-hCG level predicted the development of SGA, while the uterine artery notch in the first trimester Predicted the occurrence of PE. (3) The uterine artery notch in the first trimester demonstrated excellent negative predictive value for predicting the occurrence of PE, suggesting that subjects who had no uterine artery notch in the first trimester are highly unlikely to develop SGA or PE later.

Anatomically, the uterine arteries are the branches of the internal iliac arteries that supply most of blood to the uterus [1]. These arteries run toward the uterus and branch into the arcuate arteries that run in parallel to the superficial myometrium [1,33]. The radial arteries branch off the arcuate arteries that penetrate to the myometrium and give rise to the basal arteries and terminate as the spiral arteries that open into the intervillous space of the placenta during pregnancy [1,33]. Maternal–fetal exchanges take place in the tertiary stem villi within the placenta [33]. This remodeling of the uteroplacental vasculature occurs during pregnancy and results in decreased vascular resistance.

We found that the PI, the RI, and proportion of subjects with a notch in the uterine artery in the first trimester decreased as the gestational age increased. This is consistent with the results from previous studies, showing that both the mean uterine artery PI and the prevalence of bilateral uterine artery notches decreased as the gestational age increased from 11 to 14 weeks [34]. The mean uterine artery PI values were similar between studies except for those at 13 weeks. Of note, our values at 13 weeks were lower than that in previous studies. This difference may be due to a lower number of subjects at 13 weeks. Alves et al. also observed decreasing mean uterine artery PI and RI values as the pregnancy progressed in the first trimester [35]. These patterns may reflect the trophoblastic invasion during the first trimester, which causes a decrease in the impedance of the uterine artery and an increase in velocity and volume flow [35,36]. Difference from the uterine artery Doppler in the second trimester and varying levels of RI and PI values according to the gestational age, may prevent the accurate prediction of adverse pregnancy outcomes. Future studies with a large number of cases are required to determine at what gestational age the PI and RI values become stabilized and their predictive powers increase.

We also found that lower beta-hCG levels in the first trimester predicted the occurrence of SGA, but not PE. In contrast, previous studies showed that reduced beta-hCG levels in pregnancies were associated with subsequent development of SGA and PE [20,21]. Low levels of beta-hCG may result from decreased hormone secretion by syncytiotrophoblast of the placenta due to incomplete trophoblastic invasion. Inconsistent with our results, beta-hCG alone has been reported to be unsatisfactory as a predictive marker for SGA [7,37,38]. Moreover, we did not observe an association between the beta-hCG level and PE. The small number of cases with PE (*n* = 12) may have hindered statistical significance. Similar to beta-hCG, PAPP-A is also released by placental trophoblasts during pregnancy, thus indicating poor early placentation when PAPP-A level is low [39]. Many studies have shown an association between a low PAPP-A level and the occurrence of SGA or PE [18,20,21,38]. In this study, however, the serum PAPP-A level was not associated with SGA or PE. The discrepancy can be attributed to our exclusion of PE-positive SGA cases form the SGA group, while most previous studies did not. Since some studies have reported that serum PAPP-A rather than beta-hCG was closely associated with PE and fetal growth retardation [21], excluding PE cases from the SGA group may lead to a negative result. Different mechanisms by which beta-hCG and PAPP are lowered and why they affect pregnancy outcomes differently should be investigated.

Many studies have reported the association between the uterine artery notch and the occurrence of SGA or PE in the second trimester [4,40,41,42]. In this study, we determined that the presence of a uterine artery notch in the first trimester also significantly predicted the occurrence of PE after controlling for various confounding factors. The result was consistent with the previous study reporting that bilateral notching of uterine arteries in the first trimester predicted the development of hypertensive disorders [16]. However, no studies have so far provided adjusted odds ratio of Doppler and serum markers in the first trimester. The odds ratios for predicting PE in this study were as high as those in the second trimester in other studies [2,3,43]. The presence of a uterine artery notch in the first trimester reflects incomplete decidualization of the spiral arteries [44], which may, in turn, lead to PE. Interestingly, a unilateral uterine notch in the first trimester did not predict PE, while bilateral uterine notches predicted PE. This finding may be explained by the maintenance of sufficient blood flow through the unaffected blood vessels [45]. Given the excellent sensitivity (91.7%) and negative predictive value (99.6%), a notch in the first trimester may be a good screening tool for the prediction of PE.

Conversely, a uterine artery notch in the first trimester was not related to the presence of SGA. This may be due to the heterogeneous etiology of SGA, both placental insufficiency-related or -unrelated [7,46]. Compared to the excellent sensitivity (91.7%) of a uterine artery notch in the first trimester for PE prediction, the relatively low sensitivity (62.5%) of a uterine notch in the first trimester for SGA prediction can also be explained by the same reason. Since both the uterine PI and RI values are measured at the end of the trophoblastic invasion [10], they are less likely to change in the second trimester, and the predictive power of the PI or the RI in the second trimester for SGA and PE was significant and consistent. However, the PI and RI values change according to gestational weeks in the first trimester. Therefore, it may be necessary to define the specific PI and RI cutoff references for each gestational week and see whether and at which gestational week, the PI or RI values predict poor pregnancy outcomes.

While the presence of a uterine notch in the first trimester reflected the decidual transformation of the spiral arteries, the persistence of a notch after the second trimester reflects an abnormally high placental bed resistance [2,47]. This difference can be explained by the two steps involved in trophoblastic invasion [45]. Between 8 and 12 weeks of gestation in the first trimester, the trophoblast invades the decidual part of the spiral arteries, but only the superficial portion [2]. From 14 weeks, the trophoblast invasion progresses into the deep myometrium, thereby affecting placental resistance. In line with our Doppler finding, a pathological study reported that bilateral notching was observed in 45 of 100 cases with normal pathology in the first trimester but decreased to 12 of 100 cases in the second trimester [48].

A persistent notch was found to significantly predict later occurrence of SGA and PE. In line with our study, similar results have been reported by several other studies [40,42]. However, caution is required to interpret the results. More than half of the subjects (57.3%) had a uterine artery notch on at least one side in the first trimester, while only 8.3% of them had a uterine artery notch in the second trimester. Also, a uterine artery notch in the second trimester only was closely associated with SGA and PE, regardless of persistence. Moreover, the significance of uterine artery notch in the second trimester may drive the main effect of persistent notch. Our findings suggest that it is more important to focus on the negative predictive value of the uterine artery notch in the first trimester to predict the notch in the second trimester. When the subjects had no notch in the first trimester, 99.6% also had no notch in the second trimester. There was only subject in our study who had no notch in the first trimester but a notch in the second trimester, and the subject did not develop SGA or PE. One other study reported similar results to our study and demonstrated that none of the subjects with no notch in the bilateral uterine arteries between 11 to 14 weeks, had notches bilaterally between 18 and 23 weeks [41]. Another study showed that only 3% of the women without a notch in the first trimester had bilateral uterine artery notches between 19 and 22 weeks [2]. Interestingly, the author criticized their results due to the low reproducibility of the bilateral notch measurements in the first trimester compared to the second trimester, resulting in controversy. Carbillion et al. also reported the risk of a persistent notch in relation to the occurrence of SGA and PE. However, in that study, there was no case of a notch in the second trimester among those without a notch in the first trimester [42]. These studies suggest that the absence of a notch in the uterine artery in the first trimester may be useful because of its excellent negative predictive value for the presence of a notch in the second trimester. Considering the significant association between a notch in the second trimester and SGA and PE, screening for the absence of a uterine artery notch in the first trimester may play an important role to predict favorable pregnancy-related outcomes.

Moreover, our findings may be considered in relation to aspirin prescriptions for women at risk of PE. The American College of Obstetricians and Gynecologists (ACOG) and the Society for Maternal-Fetal Medicine recommend low-dose aspirin prophylaxis before 16 weeks for pregnant women at risk of PE [49]. However, it is difficult to screen those at risk for PE in the first trimester. Furthermore, the problem of compliance and the potential risks of taking aspirin, such as gastrointestinal and cerebral bleeding, should also be considered. Our results suggest that no uterine artery notch in the first trimester may be a good exclusion criterion for selecting subjects who would benefit from aspirin prophylaxis.

## 5. Study Limitation

This study has several limitations. First, the number of subjects enrolled in this study and the number of PE cases were small, which may prevent generalization of the results. A larger number of cases or external validation are necessary to validate the results of this study. Second, as risk factors of SGA or PE, smoking, renal disease, gestational diabetes mellitus in previous pregnancy, prior PE history, and family history of PE were not investigated completely in this retrospective study. Future studies investigating all potential risk factors for SGA and PE are required to demonstrate the independent association between the uterine artery Doppler findings in the first trimester and the development of SGA or PE. Third, cases of fetal growth restriction were not clearly distinguished from SGA. A thorough investigation of fetal growth restriction may enrich the value of Doppler findings in the first trimester. Fourth, the number of subjects who underwent Doppler in 13 weeks of gestational age was small, which would prevent finding a statistically significant changes in serum biomarkers and Doppler findings with increasing gestational ages. In future studies, it may be helpful to perform Doppler sonography in subjects from a wider range of gestation ages in the first trimester to determine the pattern of the serum biomarkers and Doppler findings more obviously. Fifth, the prevalence of SGA in this cohort was 17.5%, which was relatively higher compared with the prevalence form previous reports [50]. The high prevalence in this study may be related to this being a single tertiary center study, and thus, high-risk subjects were more likely to visit and receive prenatal care. Moreover, the maternal age in this study was older than in previous studies in other countries. Last, in terms of the negative predictive value of the uterine artery notch in the first trimester, there was only one subject without a notch in the first trimester but with a notch in the second trimester. Although the subject had neither SGA nor PE, we cannot confirm that this case would have a favorable pregnancy outcome. Studies with larger sample sizes are needed to draw more evident conclusive evidence.

## 6. Conclusions

Although there were some variations in the serum biomarkers and Doppler findings in the first trimester, lower serum beta-hCG levels and a uterine artery notch in the first trimester significantly predicted the occurrence of SGA and PE, respectively. Moreover, a uterine arterial notch in the first trimester has a high negative predictive value for predicting PE, suggesting that subjects who had no uterine artery notch in the first trimester are highly unlikely to develop SGA or PE later. This study highlights the clinical importance of assessing serum beta-hCG and the uterine artery notch in the first trimester to predict SGA and PE.

## Figures and Tables

**Table 1:** Maternal demographic characteristics, and obstetric and perinatal outcomes.

	SGA			PE		
	Absence	Presence	*p* Value	Absence	Presence	*p* Value
Number	512	104		616	12	
Age, year	34.0 ± 4.0	33.4 ± 3.1	0.066	33.9 ± 3.8	33.5 ± 2.1	0.563
Body mass index, kg/m^2^			0.278			0.134
<23	338 (66.0)	77 (74.0)		415 (67.4)	5 (41.7)	
23–25	87 (17.0)	13 (12.5)		100 (16.2)	3 (25.0)	
>25	87 (17.0)	14 (13.5)		101 (16.4)	4 (33.3)	
Parity			0.049			0.478
Nulliparous	405 (79.1)	91 (87.5)		496 (80.5)	11 (91.7)	
Parous	107 (20.9)	13 (12.5)		120 (19.5)	1 (8.3)	
Chronic hypertension	4 (0.8)	1 (1.0)	>0.999	5 (0.8)	1 (8.3)	0.110
Diabetes mellitus	9 (1.8)	0 (0.0)	0.369	9 (1.5)	0 (0.0)	>0.999
Autoimmune disease	3 (0.6)	0 (0.0)	>0.999	3 (0.5)	0 (0.0)	>0.999
Gestational hypertension	1 (0.2)	0 (0.0)	>0.999	1 (0.2)	5 (41.7)	<0.001
Gestational diabetes mellitus	40 (7.8)	5 (4.8)	0.407	45 (7.3)	2 (16.7)	0.224
Gestational age at birth, wks	38.6 ± 1.6	38.9 ± 1.8	0.094	38.6 ± 1.7	36.3 ± 1.8	<0.001
Premature delivery	26 (5.1)	4 (3.8)	0.803	30 (4.9)	6 (50.0)	<0.001
Birth weight, kg	3.3 ± 0.4	2.8 ± 0.3	<0.001	3.2 ± 0.4	2.5 ± 0.7	0.001
1 min Apgar < 7	22 (4.3)	2 (1.9)	0.260	24 (3.9)	0 (0.0)	>0.999
5 min Apgar < 7	4 (0.8)	0 (0.0)	>0.999	4 (0.7)	0 (0.0)	>0.999
Serum markers, *n*	350	83		433	10	
beta-hCG	1.2 ± 0.5	1.1 ± 0.5	0.018	1.2 ± 0.5	1.2 ± 0.3	0.787
PAPP-A	1.1 ± 0.6	1.0 ± 0.5	0.422	1.1 ± 0.6	1.1 ± 0.5	0.656

Values are expressed as the mean ± standard deviation or number (percentage). Abbreviations: beta-hCG, beta-human chorionic gonadotropin; PAPP-A, pregnancy-associated plasma protein A; PE, preeclampsia; SGA, small for gestational age.

**Table 2:** The relationship of the uterine artery notch between the first and second trimesters.

	Uterine Artery Notch in the Second Trimester		*p*-Value	Persistent Notch		*p*-Value
	Absence	Presence		Absence	Presence	
Uterine artery notch in the first trimester						
According to the presence of notch						
Absence (*n* = 268)	267 (99.6)	1 (0.4)	<0.001	268 (100.0)	0 (0.0)	<0.001
Presence (*n* = 360)	330 (91.7)	30 (9.2)		330 (91.7)	30 (9.2)	
According to the number of notches						
None (*n* = 268)	267 (99.6)	1 (0.4)	<0.001 ^a,b^	268 (100.0)	0 (0.0)	<0.001^a,b^
Unilateral (*n* = 106)	100 (94.3)	6 (5.7)		100 (94.3)	6 (5.7)	
Bilateral (*n* = 254)	230 (90.6)	24 (9.4)		230 (90.6)	24 (9.4)	

Significant difference ^a^ between the none and unilateral groups and ^b^ between the none and bilateral groups.

**Table 3:** The presence or absence of the uterine artery notch according to the presence of SGA and PE.

	SGA		*p* Value	PE		*p* Value
	Absence	Presence		Absence	Presence	
Notch, 1st, *n* (%)						
Absence	228 (44.5)	39 (37.5)	0.187	267 (43.3)	1 (8.3)	0.015
Presence	284 (55.5)	65 (62.5)		349 (56.7)	11 (91.7)	
None	228 (44.5)	39 (37.5)	0.332	267 (43.3)	1 (8.3)	0.020 ^a,b^
Unilateral	86 (16.8)	17 (16.3)		103 (16.7)	3 (25.0)	
Bilateral	198 (38.7)	48 (46.2)		246 (39.9)	8 (66.7)	
Notch, 2nd, *n* (%)						
Absence	499 (97.5)	92 (88.5)	<0.001	591 (95.9)	5 (50.0)	<0.001
Presence	13 (2.5)	12 (11.5)		25 (4.1)	6 (50.0)	
None	499 (97.5)	92 (88.5)	<0.001 ^a,b^	591 (95.9)	6 (50.0)	<0.001 ^a,b^
Unilateral	8 (1.6)	10 (9.6)		18 (2.9)	4 (33.3)	
Bilateral	5 (1.0)	2 (1.9)		7 (1.1)	2 (16.7)	
Notch group			<0.001 *			<0.001 *
UAN1st_−_2nd_−_ group	227 (44.3)	39 (37.5)		266 (43.2)	1 (8.3)	
UAN1st_+_2nd_−_ group	272 (53.1)	53 (51.0)		325 (52.8)	5 (41.7)	
UAN1st_−_2nd_+_ group	1 (0.2)	0 (0.0)		1 (0.2)	0 (0.0)	
UAN1st_+_2nd_+_ group	12 (2.3)	12 (11.5)		24 (3.9)	6 (50.0)	

UAN1st_−_2nd_−_ group indicates the group without a uterine artery notch in both the first and second trimesters. UAN1st_+_2nd_−_ group indicates the group with a uterine artery notch in the first trimester but not in the second trimester. UAN1st_−_2nd_+_ group indicates the group without a uterine artery notch in the first trimester but with a notch at the second trimester. UAN1st_+_2nd_+_ group indicates the group with uterine artery notch in both the first and second trimesters, indicating the persistent notch. Significant difference ^a^ between the none and unilateral groups and ^b^ between the none and bilateral groups. * Significant differences between UAN1st_−_2nd_−_ group and UAN1st_+_2nd_+_ group and between UAN1st_+_2nd_−_ group and UAN1st_+_2nd_+_ group. Abbreviations: PE, preeclampsia; SGA, small for gestational age.

**Table 4:** Diagnostic performance of the uterine artery notch in the first trimester to predict the occurrence of SGA and PE.

	Sensitivity (%)	Specificity (%)	PPV (%)	NPV (%)
SGA	62.5	44.5	18.6	85.4
PE	91.7	43.3	3.1	99.6

Abbreviations: NPV, negative predictive value; PE, preeclampsia; PPV, positive predictive value; SGA, small for gestational age.

**Table 5:** Odds ratios of the PI and the RI from the first Doppler sonography according to the presence of SGA and PE.

	SGA			PE		
	Adjusted OR	95% CI	*p* Value	Adjusted OR	95% CI	*p* Value
1st trimester						
PI	1.32	0.94–1.86	0.108	1.23	0.47–3.26	0.674
RI	1.11	0.26–4.79	0.884	7.70	0.63–93.60	0.109
Notch	1.21	0.77–1.90	0.421	8.65	1.06–77.77	0.045
Unilateral	1.08	0.57–2.04	0.819	7.98	0.71–90.11	0.093
Bilateral	1.26	0.78–2.05	0.350	8.91	1.05–82.28	0.047
Beta-hCG	0.53	0.27–0.90	0.019	1.06	0.28–3.93	0.935
PAPP-A	0.69	0.43–1.10	0.115	1.14	0.39–3.32	0.808
2nd trimester						
PI	3.27	1.49–7.16	0.003	16.98	3.75–76.94	<0.001
RI	15.88	1.16–216.65	0.038	661.92	23.20–237.36	<0.001
Notch	5.10	2.20–11.82	<0.001	18.21	5.12–64.76	<0.001
Unilateral	7.07	2.63–18.99	<0.001	15.18	3.52–65.53	<0.001
Bilateral	2.05	0.37–11.27	0.409	27.29	4.05–184.12	0.001
Persistent notch	5.51	2.34–12.98	<0.001	18.56	5.20–66.27	<0.001

Data are results from the logistic regression model analysis after controlling for maternal age, gestational age, chronic hypertension, autoimmune disease, gestational diabetes mellitus, nulliparity, and body mass index. Abbreviations: beta-hCG, beta-human chorionic gonadotropin; PAPP-A, pregnancy-associated plasma protein A; PE, preeclampsia; PI, pulsatility index; RI, resistance index; SGA, small for gestational age.

## Data Availability

The datasets used and/or analyzed during the current study are available from the corresponding author on reasonable request.

## Supplementary Materials

The following supporting information can be downloaded at: https://www.mdpi.com/article/10.3390/diagnostics15020233/s1, Table S1: References for the mean uterine artery pulsatility and resistance indices, and the presence or absence of the notch; Table S2: The proportion of absent, unilateral, and bilateral notches in the first and second trimesters.
