# Supplementary material for: Maternal Uterine Artery Doppler and Serum Marker in the First Trimester as Predictive Markers for Small for Gestational Age Neonates and Preeclampsia: A Pilot Study
Source: Diagnostics (Basel). 2025 Jan 20;15(2):233. doi: 10.3390/diagnostics15020233 (PMC11764443; doi:10.3390/diagnostics15020233)
Supplement: Supplementary file 1 [file diagnostics-15-00233-s001.zip › diagnostics-3395538-supplementary.pdf]

Supplementary Table S1. References for the mean uterine artery pulsatility and resistance indices, and the presence or absence of the notch

	10 weeks	11 weeks	12 weeks	13 weeks
Number	57	352	201	18
Pulsatility index				
10th percentile	1.55	1.32	1.16	1.06
50th percentile	2.28	1.92	1.80	1.59
90th percentile	2.94	2.81	2.68	2.32
Resistance index				
10th percentile	0.73	0.68	0.65	0.60
50th percentile	0.83	0.80	0.78	0.74
90th percentile	0.92	0.91	0.90	0.85
Notch				
Absence, *n* (%)	10 (17.5)	147 (41.8)	98 (48.8)	13 (72.2)
Presence, *n* (%)	47 (82.5)	205 (58.2)	103 (51.2)	5 (27.8)
None	10 (17.5)	147 (41.8)	98 (48.8)	13 (72.2)
Unilateral	15 (26.3)	58 (16.5)	31 (15.4)	2 (11.1)
Bilateral	32 (56.1)	147 (41.8)	72 (35.8)	3 (16.7)

Supplementary Table S2. The proportion of absent, unilateral, and bilateral notches in the first and second trimesters

		Uterine artery notch at the second trimester		
		None	Unilateral	Bilateral
Uterine artery notch at the first trimester	None	267 (99.6)	0 (0.0)	1 (0.4)
	Unilateral	100 (94.3)	4 (3.8)	2 (1.9)
	Bilateral	230 (90.6)	18 (7.1)	6 (2.4)

Data are expressed as number (percentage).
